# Unveiling Drimenol: A Phytochemical with Multifaceted Bioactivities

**DOI:** 10.3390/plants13172492

**Published:** 2024-09-05

**Authors:** Zhongming Yang, Kim Wei Chan, Md Zuki Abu Bakar, Xi Deng

**Affiliations:** 1 Natural Medicines and Products Research Laboratory, Institute of Bioscience, Universiti Putra Malaysia, Serdang 43400, Malaysia; yzm719268164@gmail.com (Z.Y.); chankim@upm.edu.my (K.W.C.); 2 Department of Veterinary Preclinical Science, Faculty of Veterinary Medicine, Universiti Putra Malaysia, Serdang 43400, Malaysia

**Keywords:** drimenol, phytochemical, source, bioactivity, mechanism

## Abstract

Drimenol, a phytochemical with a distinct odor is found in edible aromatic plants, such as *Polygonum minus* (known as kesum in Malaysia) and *Drimys winteri*. Recently, drimenol has received increasing attention owing to its diverse biological activities. This review offers the first extensive overview of drimenol, covering its sources, bioactivities, and derivatives. Notably, drimenol possesses a wide spectrum of biological activities, including antifungal, antibacterial, anti-insect, antiparasitic, cytotoxic, anticancer, and antioxidant effects. Moreover, some mechanisms of its activities, such as its antifungal effects against human mycoses and anticancer activities, have been investigated. However, there are still several crucial issues in the research on drimenol, such as the lack of experimental understanding of its pharmacokinetics, bioavailability, and toxicity. By synthesizing current research findings, this review aims to present a holistic understanding of drimenol, paving the way for future studies and its potential utilization in diverse fields.

## 1. Introduction

Plants are among the most versatile and proficient chemists of nature. Utilizing basic molecules, such as carbon dioxide, water, and inorganic ions, they synthesize sugars and subsequently produce an endless array of structurally diverse chemical compounds [1]. Throughout the existence of humans and animals, they have relied on plants for sustenance and various health benefits [2]. It is frequently asserted that every plant possesses medicinal properties. Holistically speaking, a medicinal plant is defined as one that contains bioactive substances (metabolites) in one or more of its organs, which can be utilized for therapeutic purposes or serve as precursors (templates) for the semi-synthesis of drug-like molecules [1]. Recent efforts have focused on developing innovative therapeutic compounds derived from natural products for various applications, such as in the agricultural, food, medicinal, pharmaceutical, cosmetic, and fragrance industries [3]. This means that finding bioactive molecules in natural products remains a great requirement. Furthermore, a shortcut to the discovery of active compounds is from medicinal and ethnopharmacological plants, especially aromatic plants [4].

Aromatic plants have been used for food, spice, and medicinal purposes since the beginning of human history [5]. Over time, they began to be used worldwide in various foods to flavor them and also for preservative purposes [6]. They are used in different forms, such as extracts, essential oils, ground leaves, or powders, to improve flavor and color, and provide antimicrobial and antioxidant effects [6]. The aroma compounds in aromatic plants are responsible for their distinctive smell and taste flavor [7]. Also, these compounds are typically stored in specialized structures, such as glands or secretory cells, and serve various ecological functions, including deterring herbivores, deterring pests, and attracting pollinators [8,9]. These compounds have diverse applications across various industries, including perfumery, aromatherapy, food, traditional medicine, pharmaceuticals, and cosmetics [10,11]. Given this, many scientists have been persistently looking for aromatic compounds with various biological activities from aromatic plants, and have made many gratifying discoveries [12,13]. For example, these aromatic compounds have anti-diabetic, hypolipidemic, antioxidant, anti-inflammatory, anti-tumor, anticonvulsant, antidepressant, anti-nociceptive, and antibiotic activities [7,14].

According to our previous review, *Polygonum minus* (PM) is a dietary aromatic plant that is frequently used as a food and flavoring agent, and is also a botanical treasure with a wide range of applications and functional properties [15,16]. By studying the composition of its phytochemicals, it was found that the compound named drimenol, presented here, was detected in its different parts, i.e., roots, stems, and leaves [17,18,19,20,21]. Moreover, in a previous article, the bioactivity of drimenol was briefly described in one sentence, including its anti-allergic, cytotoxic, insecticidal, antibacterial, antifungal, molluscicidal, piscicidal, and antifeedant activities, as well as plant growth modulation [22]. Although the biological effects of drimenol have been extensively reported, the majority of previous reports is scattered without systematic summarization [23,24,25]. In this review, we provided a comprehensive and up-to-date summary of the sources, synthesis, and derivatives of drimenol, drawing from the current literature. We emphasized the potential mechanisms underlying its reported bioactivities and discussed future application prospects. This will pave the way for a more informed exploration and utilization of drimenol, fostering its development and application.

## 2. Drimenol

### 2.1. Structure and Chemical Properties of Drimenol

**Figure 1:**
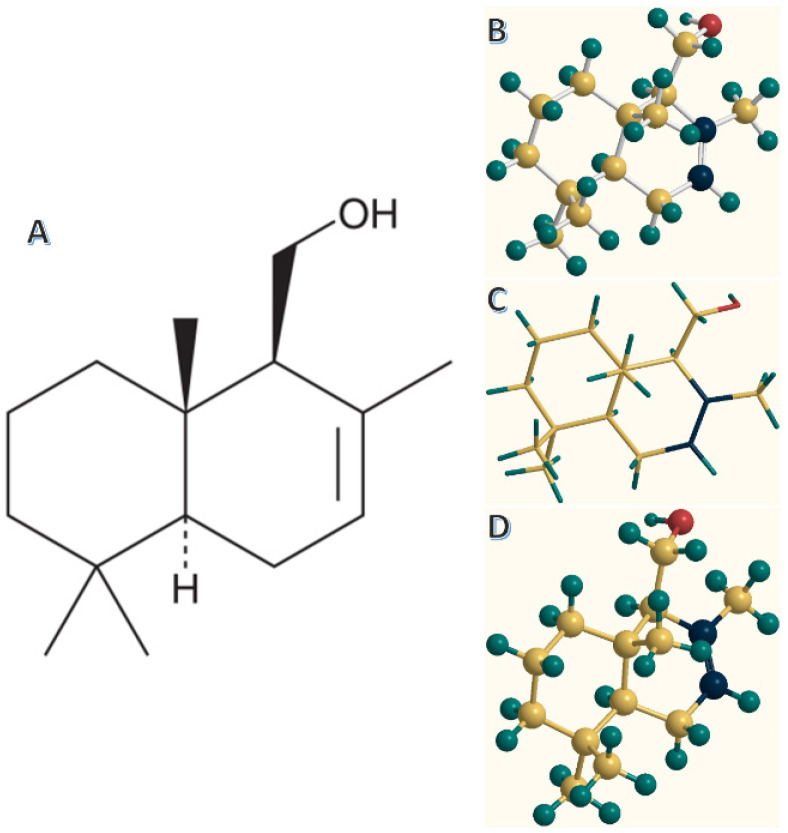
(**A**) Chemical structure of drimenol; (**B**) model of cylindrical bonds; (**C**) model of sticks; (**D**) model of ball and stick.

Drimenol is a sesquiterpene alcohol with diverse natural bioactivities [26]. Sesquiterpenoids are natural compounds with a 15-carbon frame skeleton that are considered to be important for plant protection and for humans, presenting many biological activities, such as anti-inflammatory, antioxidant, antimicrobial, antitumor, and cytotoxic properties [27,28,29]. Drimenol was first isolated in 1948 from the bark of the aromatic plant *Drimys winteri* Forst (DW), which is used as a food flavoring and pepper replacement in Argentina and Chile [30]. Its absolute configuration was identified by Appel et al. [31], with the chemical structure displayed in Figure 1. The molecular formula of drimenol is C_15_H_2_O_6_, with a molecular weight of 222.37 g/mol and an appearance of colorless to pale-yellow liquid. Its density is 0.92 g/cm^3^, boiling point is 260–262 °C at 760 mmHg, and flash point is 110 °C. It was the first bicyclic sesquiterpene with the structure and absolute configuration characteristic of the A, B ring system of many di- and triterpenes [32] and sesquiterpenoid primary alcohol, being methanol in which one of the methyl hydrogens is substituted by a 2,5,5,8a-tetramethyl-1,4,4a,5,6,7,8,8a-octahydronaphthalen-1-yl group. It is also known as [(1S,4aS,8aS)-2,5,5,8a-Tetramethyl-1,4,4a,5,6,7,8,8a-octahydro-1-naphthalenyl] methanol [ACD/IUPAC name]. This unique structure is responsible for its characteristic odor, often described as sweet, woody, and slightly earthy [33]. Drimenol is soluble in various organic solvents, such as ethanol, methanol, dimethyl sulfoxide, and dimethyl formamide. However, drimenol is insoluble in water.

### 2.2. Plant Sources of Drimenol

Drimenol, a secondary metabolite produced during plant metabolism, is widely distributed across various plants globally, with PM being the primary and most accessible source. All parts of PM (its leaves, stems, and even roots) and its essential oils are rich sources of drimenol [18,19,21,34,35,36,37,38,39]. In addition, drimenol can be isolated from other plants, as listed in Table 1.

### 2.3. Extraction, Isolation, and Characterization of Drimenol

The methods for extracting drimenol have been extensively researched and well-documented [93,125,126,127,128,129]. The conventional technique for drimenol extraction is maceration [130]. These extraction techniques are primarily simple without complex experimental configurations and are implemented on many plant materials, such as DW bark, *Bazzania trilobata*, and *Polygonum acuminatum* Kunth [131,132,133,134,135]. The extraction process typically begins with drying the plant material and grinding it into a fine powder. The solvents used for extraction include ethyl acetate, n-hexane, a mixture of ethyl acetate-n-hexane, and dichloromethane. The concentrated drimenol-containing extract is prepared by evaporating the crude extract in a rotary evaporator. Drimenol was isolated and further purified using preparative column chromatography on silica gel. Primary fractionation of the crude extract using solvents of increasing polarities from hexane to ethyl acetate was analyzed by thin-layer chromatography (TLC). A crystalline compound with different retention times on TLC was then produced and identified as drimenol by NMR.

Nevertheless, traditional extraction methods often require large amounts of solvents, longer extraction times, and are generally less efficient [136]. To address these limitations, some researchers have explored alternative methods [106]. Specifically, branches of *Drimys angustifolia* were dried in the dark at room temperature and pulverized into fine powder. The branch oils were extracted through hydrodistillation for 4 h using a modified Clevenger-type apparatus under a nitrogen atmosphere. Hexane was added to the crystallized branch essential oil from *Drimys angustifolia* in a 1:1 volume ratio. Upon heating, a homogeneous mixture was formed, which was subsequently cooled in a refrigerator for several hours, resulting in the separation of drimenol as colorless crystals [106]. It is not difficult to see that they need less extraction solvent, less extraction time, and have a higher extraction efficiency, compared with traditional approaches. Nonetheless, a specially designed experimental setup is required, which may be expensive during installation. These technologies are still in the stage that needs further development.

Once drimenol is extracted and purified from the plant material, it must be characterized to analyze its properties. The productivity of the entire extraction procedure depends on the correct identification of drimenol. Therefore, scientists used Fourier-transform infrared spectroscopy (FTIR) and nuclear magnetic resonance (NMR) and compared the results with those of pure standards by spectroscopic methods [133]. Pure compounds were identified based on the optical rotation, micro-melting point, and spectroscopic data from ^1^H and ^13^C NMR [52], and were compared with the literature data for drimenol [137].

### 2.4. Synthesis of Drimenol

#### 2.4.1. Biosynthesis of Drimenol

A decade ago, Kwon et al. [26] investigated the biosynthesis of drimenol in the roots of the valerian plant (*Valeriana officinalis*). They identified a novel sesquiterpene synthase cDNA (VoTPS3) that catalyzes the formation of drimenol from farnesyl diphosphate (FPP). NMR analyses, following the purification of the terpene produced by VoTPS3 and the characterization of the VoTPS3 enzyme, confirmed that VoTPS3 synthesizes drimenol. Regarding the mechanism of drimenol synthesis, the researchers suggested that drimenol synthase (DMS) might utilize protonation-initiated cyclization. These findings indicate that VoTPS3 can be used to produce drimenol in plants [26].

In a separate study, researchers identified and characterized both a DMS and a cytochrome P450 drimenol oxidase (PhDOX1) from *Persicaria hydropiper*, which is involved in the biosynthesis and conversion of drimenol. The expression of DMS alone resulted in the production of drimenol, whereas the co-expression with PhDOX1 primarily yielded drimendiol and cinnamolide in yeast [138]. These results highlight the critical role of DMS in drimenol production.

**Figure 2:**
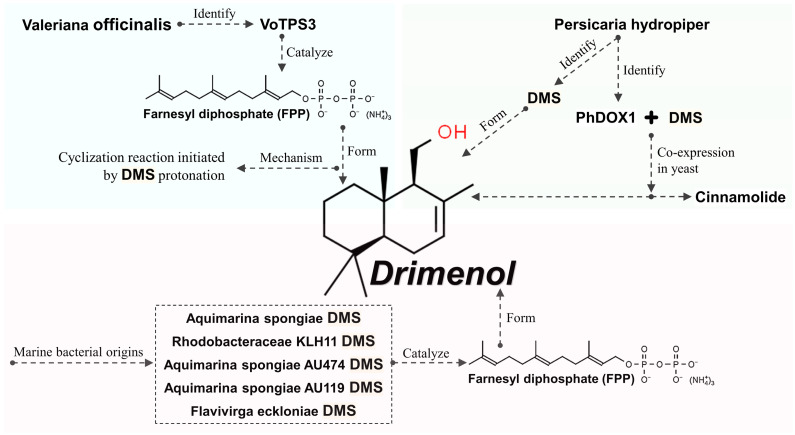
Biosynthesis pathways of drimenol.

Consequently, other researchers have focused on DMS, discovering five DMSs of marine bacterial origin [24]. These include two recombinant proteins (*Aquimarina spongiae* DMS and Rhodobacteraceae KLH11 DMS) and three candidates (*Aquimarina spongiae* AU474 DMS, *Aquimarina spongiae* AU119 DMS, and *Flavivirga eckloniae* DMS), all of which catalyze the biosynthesis of drimenol from FPP [24]. The overall biosynthesis mechanism of drimenol is illustrated in Figure 2.

#### 2.4.2. Chemical Synthesis of Drimenol

**Figure 3:**
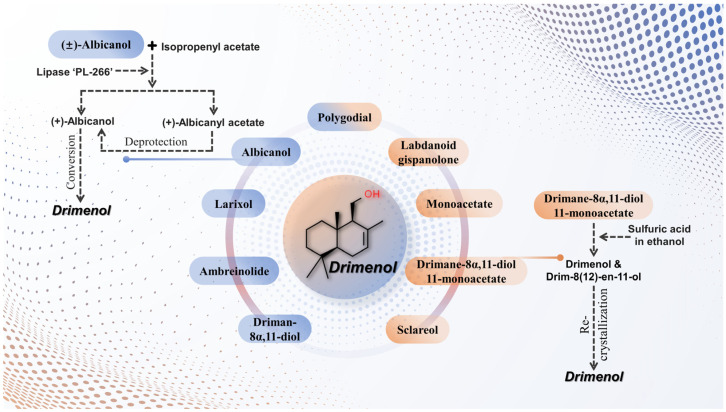
Chemical synthesis of drimenol.

Besides biosynthesis pathways, drimenol can be obtained by chemical synthesis. The relatively low content of drimenol in natural sources has spurred studies on its synthetic production [139]. Several methods for drimenol synthesis have been developed (Figure 3). For instance, Akita et al. [140] used lipase ‘PL-266’ from *Alcaligenes* sp. to perform the enantioselective acetylation of albicanol with isopropenyl acetate, resulting in enantiomerically pure albicanyl acetate and albicanol. Subsequent deprotection of albicanyl acetate produced natural albicanol, which was then converted into drimenol [140].

A convenient and efficient method for drimenol synthesis starting from drimane-8α,11-diol 11-monoacetate was proposed by Kuchkova et al. [141]. This process is highlighted due to its mild conditions and good yield. The key step involves treating drimane-8α,11-diol 11-monoacetate with sulfuric acid in ethanol. The reaction proceeds under mild conditions (20 °C for 18 h), yielding a mixture of drimenol and its isomer drim-8(12)-en-11-ol in approximately a 10:1 ratio. The mixture is then recrystallized from hexane to obtain pure drimenol, with a yield of 52.8%, and its physicochemical properties match those of an authentic sample [141].

Additionally, Aricu [139] summarized several compounds that can be used for drimenol synthesis, including ambreinolide, labdanoid gispanolone, larixol, sclareol, monoacetate, and driman-8α,11-diol [139]. In another study, Rihak et al. [142] successfully isolated gram-scale quantities of highly pure polygodial from *Tasmannia lanceolata* in a few hours. They employed polygodial for the semi-syntheses of several structurally related natural products, including drimenol [142,143].

## 3. Biological Activities of Drimenol

As mentioned above, drimenol possesses various biological activities. The elucidation of its biological activities has aroused huge interest in the scientific community. Numerous research reports published to date have explored the varied beneficial biological activities and applications of drimenol. In this section, we will focus on the general biological activities and applications of drimenol, as well as provide a brief description of its mechanism of action.

### 3.1. Antifungal Activity of Drimenol

**Figure 4:**
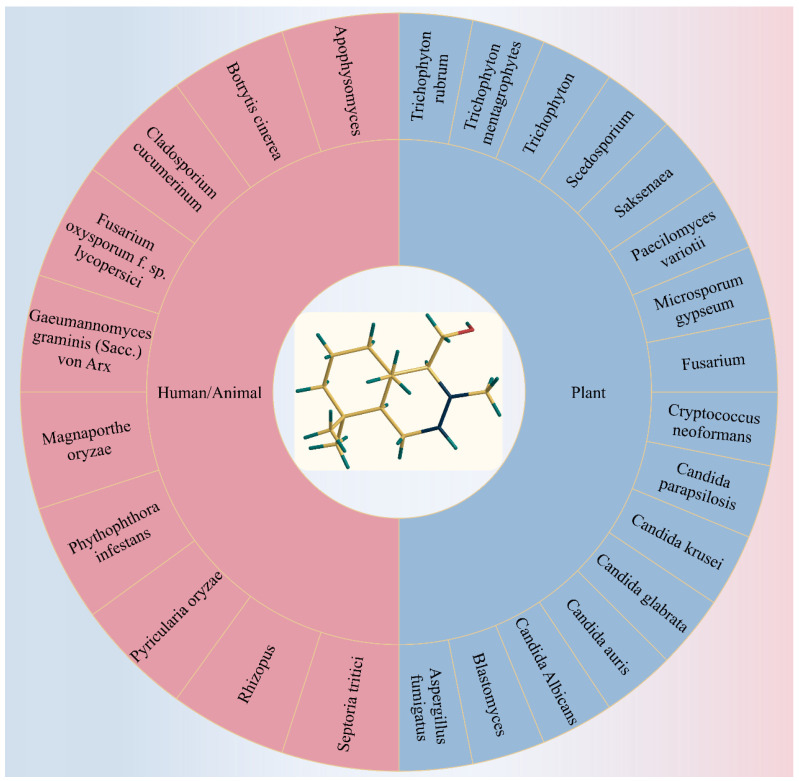
Antifungal activity of drimenol.

Drimenol is a broad-spectrum antifungal agent effective against a wide range of pathogenic organisms, as illustrated in Figure 4. Previous studies have reported its antifungal activity against various pathogenic fungi. It has been determined that the presence of a Δ7,8-double bond in the drimane skeleton of drimenol is a critical structural feature for its antifungal efficacy. Additionally, the aldehyde group at C-9 appears to be non-essential for its activity [127]. To have a comprehensive understanding of its antifungal potential, we summarized the effects of drimenol on various fungi pathogenic to humans, animals, and plants, as well as its mechanisms of action in several fungal species.

#### 3.1.1. Phytopathogenic Fungi

The agricultural industry places significant emphasis on managing plant diseases due to the substantial economic and biosecurity risks posed by plant pathogens [144]. Among these, fungal infections are particularly threatening to food production [145], evidenced by historical events, like the Irish Potato Famine, and contemporary challenges, such as rice blast and wheat rust, which jeopardize food security and incur significant economic losses [146]. Although conventional fungicides have played a crucial role in enhancing food security and controlling agricultural diseases, their associated risks have prompted the exploration of alternative fungal control methods [147]. These alternatives should ideally be environmentally friendly and have minimal adverse effects on animal health when applied exogenously. Over millions of years, plants have evolved diverse defense mechanisms against fungal infections, providing biodegradable, generally non-toxic solutions antagonistic to harmful microorganisms [148]. In this section, we summarize and discuss drimenol as a promising candidate for the future development of antifungal agents for agricultural purposes, as depicted in Figure 4.

The effects of drimenol on the mycelial growth of *Botrytis cinerea* were evaluated by Robles-Kelly et al. [149]. The results indicated that drimenol inhibited the growth of *Botrytis cinerea* with an EC50 value of 80 ppm. Moreover, at concentrations of 40 and 80 ppm, the germination rate of *Botrytis cinerea* was reduced to nearly half of the control value [149,150,151].

Monsálvez et al. [131] investigated the effects of an n-hexane extract from the bark of DW on wheat seedlings inoculated with *Gaeumannomyces graminis* var. tritici. The study found that a dose of 250 mg/kg of the n-hexane extract effectively controlled *Gaeumannomyces graminis*, resulting in significantly greater plant height, biomass, chlorophyll content, and stomatal conductance compared to the inoculated control, while also markedly reducing disease severity. Chemical fractionation and analysis of the n-hexane extract revealed that the antifungal activity was primarily associated with compounds such as polygodial, drimenin, drimenol, and isodrimenol. Consequently, the application of a complex mixture of these components can reduce fungal damage severity and protect the growth of wheat seedlings infected by *Gaeumannomyces graminis* [131,151,152,153].

Scher et al. [134] found that drimenol exhibited moderate antifungal activity against *Septoria tritici* (IC50: 80.1 μg/mL) and strong activity against *Cladosporium cucumerinum* (IC50: 6.6 μg/mL). Furthermore, they detected the weak activity of drimenol against *Botrytis cinerea* and *Pyricularia oryzae* [134,154,155,156,157,158,159,160].

To develop new antifungal agents based on drimane sesquiterpenes, Edouarzin et al. [161] investigated the antifungal activity of synthetic drimane terpenes, drimenol, and albicanol, along with six analogs. Their results demonstrated that drimenol is a potent fungicide and caused 100% death of various fungi at concentrations of 8–64 µg/mL, such as *Rhizopus* and *Apophysomyces* [161,162].

The ethyl acetate crude extracts of endophytic fungi isolated from *Litsea petiolata* leaves were evaluated for their antifungal activity against two isolates (THL084 and THL861) of *Magnaporthe oryzae*, the causative agent of rice blast disease determined by Pripdeevech et al. [163]. In a disc diffusion assay, the crude extract from *Fusarium* sp. MFLUCC16-1462 demonstrated antifungal activity against the THL084 isolate. In addition, after 96 h dual cultures with *Fusarium* sp. MFLUCC16-1462, the mycelium growth of THL084 and THL 861 was inhibited by 61.96% and 31.74%, respectively. Moreover, the major components of Litsea petiolate crude extracts were pregeijerene B, callitrin, drimenol, and angustione [163].

Among the pathogens that significantly impact global tomato production are *Clavibacter michiganensis* subsp. michiganensis and *Pseudomonas syringae* pv. tomato, responsible for bacterial canker and bacterial speck, respectively; the fungus *Fusarium oxysporum* f. sp. lycopersici, which induces *Fusarium* wilt; and *Phytophthora* spp., which affects both potato and tomato cultivation. Montenegro et al. [126] studied the effects of drimenol against these four phytopathogenic microorganisms. Most promisingly, study results displayed that drimenol presented inhibition activity against *Fusarium oxysporum* f. sp. lycopersici, with MIC and MFC values in the range of 128–256 µg/mL [126,164].

#### 3.1.2. Human or Animal Mycoses

In recent years, the risk of opportunistic mycotic infections has increased in immunocompromised patients, such as those patients receiving organ transplantation, cancer chemotherapy, and with human immunodeficiency virus [165,166]. In immunocompromised patients, the fungus most often causing these infections is *Candida albicans*, which causes 90% of *candidal vaginitis* in these patients and healthy women [167]. Despite great advances in drugs to treat mycotic infections, their use is limited by their side effects and the growing resistance of *Candida albicans* to antifungal drugs [168]. Amphotericin B, considered the “gold standard” antifungal drug, is extensively used for treating severe fungal infections. Nevertheless, its use can lead to nephrotoxicity and infusion-related adverse reactions [169]. Additionally, azole antifungal mediations can generate resistant strains of *Candida* species. Studies have shown that the resistance rate to fluconazole in isolates collected from women with *candidal vaginitis* ranges from 3.6% to 7.2% [170]. Moreover, surveys in the United States indicate that approximately 2 million people experience fungal and bacterial infections annually, with 65% of these patients showing resistance to at least one antimicrobial drug [171]. The continuous use of antibiotics and inadequate infection control measures have contributed to instances of drug failure in treating fungal infections [172]. Consequently, there is an increasing need for research into alternative anti-infective therapies and the development of new treatments [173,174].

Medicinal plants, long used in traditional medicine systems to treat fungal infections in humans and animals, are considered valuable sources for discovering new antifungal drugs [175]. Therefore, natural products show great potential in the discovery of new antifungal drugs [176]. Drimenol, a sesquiterpenoid primary alcohol derived from natural sources, offers a promising foundation for the development of novel antimycotic agents [177].

M. G. Derita et al. [135] conducted a study to assess the antifungal properties of the aerial parts of *Polygonum acuminatum* to validate its traditional use as an antifungal agent and to isolate the compound(s) responsible for its antifungal activity. The study revealed that drimenol was effective against Trichophyton rubrum, *Microsporum gypseum*, and Trichophyton mentagrophytes (MIC = 62.5 μg/mL), but showed no activity against *Aspergillus* spp, Saccharomyces cerevisiae, and *Candida albicans* [135,178].

Edouarzin et al. [161] synthesized drimenol from sclareolide and evaluated its antifungal activities. The results demonstrated that drimenol possessed a strong inhibitory effect on *Candida albicans*. It not only had a bactericidal effect on *Candida albicans* (MIC: 32 μg/mL), but also inhibited other fungi, such as *Paecilomyces variotii* (MIC: 16 μg/mL), *Cryptococcus neoformans* (MIC: 8 μg/mL), *Aspergillus fumigatus* (MIC: 8 μg/mL), *Fusarium* (MIC: 32 μg/mL), *Scedosporium* (MIC: 16 μg/mL), *Saksenaea* (MIC: 4 μg/mL), *Blastomyces* (MIC: 4 μg/mL), fluconazole-resistant strains of *Candida parapsilosis* (MIC: 32 μg/mL), *Candida* krusei (MIC: 30 μg/mL), *Candida glabrata* (MIC: 30 μg/mL), *Candida albicans* (MIC: 30 μg/mL), and *Candida auris* (MIC: 50 μg/mL). These findings suggest that drimenol is a broad-spectrum antifungal compound. Furthermore, at concentrations increasing up to 100 μg/mL, drimenol caused the cell wall/membrane disruption of fungi, such as *Candida albicans* and *Cryptococcus* spp. [161].

Moreover, *Candida auris* is an emerging multidrug-resistant strain associated with nosocomial infections, and it has been increasingly reported worldwide [179]. Bioscreen-based growth curve monitoring of drimenol indicated that it had superior activity compared to fluconazole, suggesting its potential usefulness against *Candida auris* and other drug-resistant fungal pathogens. Since drimenol is active against antifungally resistant strains of *Candida auris*, *Candida albicans*, and certain *Cryptococcus neoformans*, and the mechanism of action of drimenol is different from fluconazole or other clinical antifungal drugs, drimenol could be a useful additional antifungal drug with a novel target. Drimenol demonstrated synergistic activity with fluconazole (FICI < 0.5) against *Candida albicans* in a checkerboard assay, indicating its potential in combination antifungal therapies [161,180,181].

To elucidate the main characteristics required for drimenol to exhibit antifungal activity, M. Derita et al. [127] tested its efficacy against a unique set of nine fungal strains using standardized methods. The results showed that drimenol was moderately active against *Cryptococcus neoformans* (MIC100: 125 μg/mL), *Microsporum gypseum* (MIC100: 62.5 μg/mL, MFC100: 125 μg/mL), Trichophyton rubrum (MIC100: 62.5 μg/mL, MFC100: 125 μg/mL), and Trichophyton mentagrophytes (MIC100: 62.5 μg/mL, MFC100: 125 μg/mL). They also observed that the presence of aldehydes at C-9 or C-8, or a CH_2_OH group at C-8, was not necessarily required for activity. The Δ7,8-double bond within the drimane skeleton was considered a crucial structural feature for antifungal activity. The fact that both structural types of drimane exhibited antifungal properties strongly indicated that their mechanism of action did not involve a Michael addition. Moreover, the electronic properties of drimanes significantly influenced their antifungal behavior, suggesting that the electronic distribution surrounding the Δ7,8 was pivotal for activity [127].

#### 3.1.3. Antifungal Mechanisms of Drimenol

Fungal cells possess distinct structures, such as cell walls, cell membranes, and nuclei, which antifungal agents can target to inhibit their functions or directly kill fungi [182]. Currently, several antifungal drugs, such as itraconazole, voriconazole, ketoconazole, and fluconazole, are available. These drugs are sometimes used in combination with amphotericin B to treat infections caused by *Candida* species and ringworm. Nevertheless, these drugs are primarily synthetic or semi-synthetic and, despite their efficacy against fungi, can also harm normal human cells [183]. In contrast, certain natural products offer advantages over synthetic compounds. They often exhibit specific binding to fungal targets and have reduced toxicity to human cells. Natural products can target fungal cell walls, cell membranes and various organelles, disrupting internal processes and impeding fungal cell reproduction [184]. Additionally, the structural optimization of natural products has enhanced their antifungal efficacy, making them competitive with synthetic drugs. Drimenol, a key biosynthetic precursor of various naturally occurring drimane sesquiterpenes, is a potent broad-spectrum antifungal drug [24]. Therefore, this section summarizes the natural antifungal product drimenol with therapeutic efficacy and its molecular targets.

**Figure 5:**
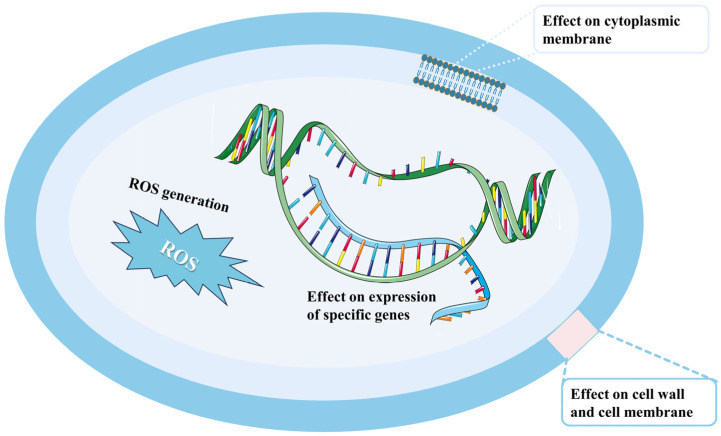
Proposed antifungal mechanisms of drimenol.

Understanding the mechanism of action of a substance is crucial for predicting potential side effects, anticipating the development of resistance, and guiding the synthesis of novel bioactive compounds [185]. The antifungal mechanisms of drimenol have been extensively studied, as illustrated in Figure 5.

##### Cytoplasmic Membrane

The plasma membrane of fungi plays a critical role in cell morphogenesis, viability, and pathogenicity [186]. Membrane damage can result in the efflux of cytoplasmic molecules, which in turn can cause fungal cell death [187]. Numerous plant bioactive compounds produce cytotoxicity through targeting the fungal plasma membrane. Notable examples include *Thymus vulgaris* CT thymol EOs, star anise EOs, tea tree, Palmarosa, esterified p-coumarates, tea tree oil, hinokitiol, *mentha piperita*, *mentha spicata*, cuminic acid, *Thymus vulgaris* CT carvacrol, and the phenanthroindolizidine alkaloid antofine [188,189,190,191,192,193,194,195]. Robles-Kelly et al. [149] used a SYTOX Green uptake assay to assess whether drimenol affects the plasma membrane integrity of *Botrytis cinerea* [196]. SYTOX Green is a high-affinity nucleic acid dye that is impermeable to the membrane of live cells, but readily penetrates cells with compromised plasma membranes [197]. Their findings showed that methanol, used as a negative control, did not result in fluorescence in the hyphal nuclei. In contrast, ethanol (70% *v*/*v*), which causes cell membrane dehydration, served as a positive control, with fluorescent nuclei observed, indicating membrane disruption. Drimenol treatment at concentrations of 40 and 80 ppm for 1 h resulted in clear fluorescence in the conidia of *Botrytis cinerea*. These results indicate that drimenol disrupts the plasma membrane of *Botrytis cinerea*, increasing its permeability to SYTOX Green and suggesting that drimenol inhibits fungal growth by compromising membrane integrity [149,196].

##### Reactive Oxygen Species (ROS) Metabolism

ROS is essential for fungi development. Nonetheless, excessive ROS accumulation can lead to irreversible oxidative damage to cellular components, including DNA, lipids, and proteins [198]. Growing evidence suggests that oxidative damage modulated by ROS is associated with the antifungal activity of plant bioactives [199]. Robles-Kelly et al. [149] examined the effects of drimenol on ROS production by incubating *Botrytis cinerea* conidia in the presence of drimenol at 21 °C for 2 h. They used the ROS-Glo™ hydrogen peroxide (H_2_O_2_) assay, a rapid, homogeneous, and sensitive luminescent assay that directly measured the levels of the H_2_O_2_ in cell cultures, to assess ROS production [200]. The results showed that drimenol significantly increased luminescence, thereby increasing ROS production [149].

##### Expression of Specific Genes

A study identified that the protective effect of drimenol against *Botrytis cinerea* is linked to the expression levels of specific genes associated with cellular damage [149]. To elucidate the mechanism of action of drimenol, Robles-Kelly et al. [149] investigated the expression changes in genes related to cellular damage, focusing on bchex, which encodes a key protein of the Woronin body, and bcnma and cas-1, which are involved in programmed death cells (PDCs). Bioinformatics analysis revealed these genes in *Botrytis cinerea*. The cas-1 gene is an ortholog of cas-A, corresponding to a metacaspase, while bcnma, a homolog of the yeast NMA11 gene, belongs to the high-temperature-requirement (HtrA) family of serine proteases and is homologous to the human HtrA2/Omi, a mitochondrial protein with pro-apoptotic functions [201]. The expression of bchex increases in response to hyphae damage, as the Woronin bodies appear to occlude septal pores within minutes [202,203]. Moreover, the gene bcaox1, which is related to oxidative stress in fungi, was analyzed [204,205]. The results showed that, in the presence of drimenol, there was no increase in the transcript levels of genes associated with PCD, suggesting that drimenol negatively modulates their transcription. However, bchex transcripts increased in the presence of drimenol compared to control conditions, indicating hyphal damage. Consistent with these findings, bcaox1 expression was also elevated in the presence of drimenol, indicating mitochondrial dysfunction. This enzyme can accept electrons from the ubiquinone pool and directly reduce oxygen, particularly when complexes III and IV in the inner membrane are impaired [204,205].

##### Cell Wall and Cell Membrane

The cell wall is vital for pathogenic fungi, serving as a permeability barrier and playing a key role in survival, adaptation, and signaling under stress during infection [206,207]. The ability to grow filamentously is essential for biofilm formation, with hyphae contributing to the structure integrity and multilayered architecture of mature biofilms [208,209]. Hyphae are significant in the pathogenicity of *Candida albicans* [210,211,212]. Research has shown that *Candida albicans* can proliferate in either yeast or hyphae forms, with the hyphae form exhibiting greater virulence [213,214]. CRK1, a member of the Cdc2 kinase subfamily, is crucial for hyphal development [215]. The deletion of CRK1 severely impaired hyphal formation under various inducing conditions, whereas the ectopic expression of its catalytic domain enhanced hyphal colony formation, even in conditions conducive to yeast growth [216]. Thus, the CRK1/CRK1 null mutant showed significant deficits in filamentous growth and the transcriptional activation of hyphal-specific genes, highlighting the importance of CRK1 in *Candida albicans* virulence [217]. To understand the broad-spectrum antifungal potential, Edouarzin et al. [161] evaluated the effects of drimenol against several human pathogenic fungi and analyzed its mechanisms of action in Saccharomyces cerevisiae and *Candida albicans*. Their yeast mutant screening and spot assay results indicated that drimenol functioned as a fungicidal agent by disrupting cellular processes related to protein trafficking between the Golgi apparatus and the ER, protein secretion (Sec system), and cell signaling, potentially involving CRK1.

Genetic methods have been employed to analyze the mechanisms of action of antifungal compounds through drug-induced hypersensitivity assays [218,219]. Edouarzin et al. [161] employed a similar methodology to demonstrate that drimenol inhibited *Candida albicans* heterozygous mutants of CDC37, Orf19.4382, Orf19.1672, and Orf19.759—known or putative targets of CRK1 kinase targets—at sub-MIC concentrations [161]. Based on Nelson [220], Cdc2 kinase (of which CRK1 is a member) plays a critical role in regulating retrograde membrane transport from the Golgi to the ER during mitosis, either independently or in conjunction with another kinase, such as MEK1. It is speculated that drimenol may disrupt the interaction between CRK1 and one or more of these gene products [220].

Current research on drimenol highlights its broad-spectrum antifungal activity and potential therapeutic applications, yet significant gaps remain. While drimenol shows promise against various fungal pathogens, including both phytopathogens and human mycoses, the precise molecular mechanisms of its action are not fully understood. Future research should focus on elucidating precise molecular mechanisms of drimenol, its interactions with fungal cellular pathways, and its potential synergies with existing antifungal agents. Additionally, studies could investigate the structural modifications of drimenol to enhance its efficacy and reduce resistance, aiming to optimize its application in both agricultural and clinical settings.

### 3.2. Antibacterial Activity of Drimenol

The proliferation of bacterial infections poses a significant threat to human life globally. Bacterial diseases represent a foremost concern for human health, ranking as the second leading cause of death as early as 2019 [221]. In recent years, antibiotic resistance has emerged as a primary concern associated with microbial infections. The World Health Organization has emphasized that infections such as pneumonia, septicemia, and foodborne illnesses are increasingly challenging to treat due to this phenomenon [222]. Since the introduction of antibiotics in the 20th century [223], bacteria have developed defense mechanisms that reduce or completely negate the effectiveness of these drugs. Consequently, there is an urgent imperative to explore complementary or alternative approaches to traditional infection treatment protocols. Numerous studies have demonstrated that compounds derived from plants in specific groups exhibited significant antibacterial activity, which can be highly beneficial [224,225,226,227].

**Figure 6:**
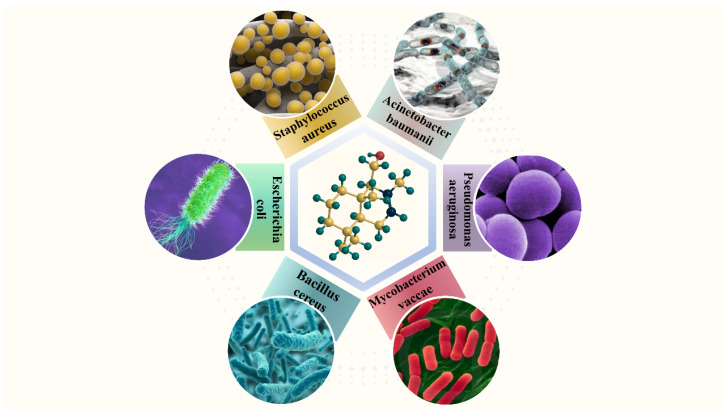
Antibacterial activity of drimenol.

Drimenol is one of the most promising sources of bioactive compounds showing antibacterial activity [228,229]. Drimenol and its derivatives have been shown to have antibacterial activity in various studies, such as against *Staphylococcus aureus*, *Pseudomonas aeruginosa*, and *Mycobacterium vaccae* [25]. Several approaches have been employed to assess the potential of drimenol as an antibacterial agent (Figure 6).

The research results of Santos et al. [106] demonstrated that drimenol exhibited MIC values of 67 µg/mL, 1333 µg/mL, 583 µg/mL, 667 µg/mL, and 667 µg/mL against *Pseudomonas aeruginosa*, *Escherichia coli*, *Acinetobacter baumanii*, *Bacillus cereus*, and *Staphylococcus aureus*, respectively. This indicates that drimenol possesses moderate antibacterial activity against *Pseudomonas aeruginosa*, *Acinetobacter baumanii*, *Bacillus cereus*, and *Staphylococcus aureus* [106,162].

Moreover, certain herbal plants, such as *Drimys granadensis* and *Polygonum hydropiper*, which contain drimenol, are widely used as antibacterial agents. Numerous studies have revealed that drimenol exhibits significant antibacterial effects, even though it is present at varying levels.

Tuberculosis is part of a group of infectious diseases responsible for approximately 90% of global deaths [230]. *Mycobacterium tuberculosis*, the bacterium causing tuberculosis, infects around eight million new individuals annually and causes a death every 10 s. Despite the designation of tuberculosis by the World Health Organization as a global health emergency, challenges persist, including prolonged treatment durations, limited diagnostic access, and the presence of multidrug-resistant strains of *Mycobacterium tuberculosis* [231]. Moreover, there has been a notable increase in infections caused by non-tuberculous *mycobacteria*, such as *Mycobacterium kansasii* and *Mycobacterium avium*, which can affect the lungs, lymphatic system, skin, and joints, leading to severe complications if untreated [232]. Therefore, discovering new active molecules targeting *mycobacteria* is of urgent importance. Alves et. al. [233] examined the antimycobacterial properties of 18 commercially available plant-derived essential oils by evaluating their efficacy against *Mycobacterium kansasii*, *Mycobacterium avium*, and *Mycobacterium tuberculosis* through MIC measurements. The majority of these essential oils exhibited minimal to no activity against these *mycobacteria*, with MIC values ranging from 1000 to 2000 µg/mL [233]. However, *Amyris balsamifera* demonstrated the highest activity against *Mycobacterium kansasii*, with an MIC of 250 µg/mL. Subsequent gas chromatography-mass spectrometry (GC-MS) analysis of *Amyris balsamifera* revealed that its major constituents were the sesquiterpenes 7-epi-eudesmol (23.6%), agarospirol (14.0%), eudesmol (12.3%), hedycaryol (10.9%), and drimenol (5.3%) [234].

The antibacterial properties of *Drimys granadensis* leaf essential oil were assessed against eight bacteria strains, including three Gram-negative and five Gram-positive strains. The results indicated that the Gram-negative bacteria (*Escherichia coli*, *Pseudomonas aeruginosa*, and Salmonella enteritidis) exhibited no sensitivity to the essential oil. Among the Gram-positive bacteria, *Staphylococcus aureus* CAMP (+), *Staphylococcus epidermidis*, *Bacillus cereus*, and multiresistant *Staphylococcus aureus* were sensitive to the oil. *Staphylococcus epidermidis* was the most sensitive, showing the widest inhibition zone (19 mm), although the zone was not as well-defined as that for *Bacillus cereus* (16 mm). Listeria monocytogenes did not exhibit an inhibition zone and was the only Gram-positive bacterium tested that was resistant to the oil. Thus, *Drimys granadensis* essential oil demonstrated antibacterial activity against half of the tested bacteria with varying degrees of effectiveness. The chemical composition of *Drimys granadensis* essential oil, obtained by the hydrodistillation of the leaves, was analyzed using GC and GC/MS, identifying 85 components. The major compounds were germacrene D (14.7%), sclarene (9.5%), α-cadinol (7.3%), longiborneol acetate (6.3%), drimenol (4.2%), (Z)-β-ocimene (4.2%), α-pinene (3.2%), and β-elemene (2.7%) [112].

Kipanga et al. [235] found that the biofilm inhibitory concentration (BIC) of drimenol required to inhibit 50% of developing biofilms in *Staphylococcus aureus* and *Staphylococcus epidermidis* was 14.7 ± 2 μg/mL and 16.4 ± 3 μg/mL, respectively [235].

*Proteus mirabilis*, a common cause of urinary tract infections, especially among elderly individuals with catheters, is the second most prevalent cause of such infections after *Escherichia coli* [236]. In the current study, the hexane fraction of *Polygonum hydropiper* exhibited the highest activity against *Proteus mirabilis*, generating inhibitory zones of 28 mm at a concentration of 100 μg/mL in the disc diffusion assay. In the well-diffusion assay, the hexane fraction produced an inhibition zone of 25 mm at the same concentration. Furthermore, the *Polygonum hydropiper* hexane fraction demonstrated significant activity, with an MIC of 100 μg/mL. GC followed by GC-MS analyses identified 124 compounds in the hexane fraction of Polygonum hydropiper, with the most abundant being 9,12,15-octadecatrienoic acid, drimenol (7.26%); methyl palmitate (7.68%), caryophyllene oxide (7.7%), methyl ester (8.85%), and humulene oxide (13.79%) were the compounds found abundantly [96].

Drimenol shows promise as an antibacterial agent, yet significant research gaps remain. Its precise antibacterial mechanisms are not fully understood, and its effectiveness against multidrug-resistant strains is underexplored. Future studies should focus on uncovering its molecular targets, evaluating its synergy with current antibiotics, and assessing its safety and pharmacokinetics to better establish its therapeutic potential.

### 3.3. Anti-Insect Activity of Drimenol

According to [237], plants have evolved various defense mechanisms to protect themselves from natural enemies. In response, pests have developed strategies to overcome these defenses. Plants typically produce compounds known as allelochemicals, which serve as protective agents against predators and microbes. These allelochemicals also help defend against vertebrates, given the similarity in neuronal signaling pathways across the animal kingdom. Moreover, combinations of secondary metabolites may offer more sustained protection against herbivores and pests than individual compounds.

Synthetic pesticides have long been employed as a prominent method for pest control. However, chemical pesticides, such as methyl bromide, phosphine, ethane dinitrile, sulfuryl fluoride, ethyl formate, and carbonyl sulfide, are associated with adverse effects on human health and the environment. Consequently, botanical products have garnered significant interest as potential alternatives. In the search for bio-insecticides, drimenol has been extensively studied as a promising substitute for conventional insecticides.

The effectiveness of insecticides varies across insect species, depending on the physiological traits of the insects and the type of insecticidal plant used. The components of various botanical insecticides can be classified into six categories: attractants, chemosterilants, growth retardants, toxicants, feeding deterrents/antifeedants, and repellents [238].

#### 3.3.1. Toxicant

Insect toxicants are substances that are harmful or lethal to insects upon exposure, leading to their incapacitation or death [239]. Some researchers found that drimenol was toxic and caused death to insects [128,132,240].

Cereals serve as a primary source of dietary protein for humans [241]; however, they are frequently infested by various stored food pests, mainly Coleoptera. These infestations lead to both quantitative and qualitative losses and can adversely affect food safety [242]. Cereal losses during storage can reach up to 50% of total production [243]. Effective grain handling and storage practices are essential to mitigate damage caused by insects, especially the wheat weevil, *Sitophilus granarius* L. (Coleoptera: Curculionidae), a persistent and destructive pest of stored grains. This insect significantly impacts the quality and yield of maize, wheat, and rice. Although several chemical fumigants with a broad spectrum of activity are employed to combat stored food pests, concerns about their adverse effects persist. These concerns include pesticide residues, pest resistance, ozone depletion (especially from halogenated fumigants), toxicity to non-target organisms, and environmental contamination [244,245]. Consequently, there is growing interest in discovering new bioactive compounds to address insect infestations. Plant-derived natural products are often preferred over traditional fumigants due to their low toxicity and biodegradability.

Paz et al. [132] assessed the dose-dependent toxicity of drimenol against *Sitophilus granaries*. They assessed acute mortality rates by incorporating varying concentrations of drimenol into the diet over six days, ranging from 0.25% to 3% (*w*/*w*). At the highest concentration, drimenol exhibited complete insect mortality within six days, with an LC50 value of 0.31% (*w*/*w*). Grain damage analysis after six days revealed that grains treated with 0.5% (*w*/*w*) drimenol experienced fewer attacks (13% incidence) compared to those treated with 0.25% (*w*/*w*) drimenol (15% incidence) [132]. This trend was consistent with the emergence of new insects relative to the initial population after 21 days. Specifically, grains treated with 0.5% (*w*/*w*) drimenol showed a 7% emergence rate of new insects, compared to a 9% emergence rate in those treated with 0.25% (*w*/*w*) drimenol. These findings highlight the potential of drimenol as a promising candidate for developing more effective derivatives against storage pests [240].

*Drosophila melanogaster*, commonly known as the vinegar fly or fruit fly, is a small dipterous insect typically measuring 3–5 mm long, with yellow–red coloring and red eyes. This insect possesses a rapid development cycle, thriving on access to sugary liquids. Its primary impact lies in the transmission of diseases such as sour rot, identifiable by the distinct vinegar odor emanating from contaminated substances. Montenegro et al. [128] assessed mortality over 168 h and determined EC50 values within the initial six days through no-choice test experiments (insecticidal bioassay) targeting first instar larvae of *Drosophila melanogaster*. Surviving larvae subsequently underwent pupation and emerged as normal insects. The larvicidal test lasted for six days, as the biological cycle from larva to pupa in *Drosophila melanogaster* takes seven days. The results showed that drimenol presented moderate activity, with an EC50 of >100 mg/L and 15.0% mortality over 168 h [128].

#### 3.3.2. Feeding Deterrents/Antifeedants

In plants, certain compounds can provoke a range of insect behavioral responses, ranging from stimulating to deterring. Deterrents, also known as antifeedants, often indicate the unsuitability of a plant by inhibiting or disrupting feeding. These compounds render treated plant materials unattractive or unpalatable, leading insects to avoid these materials and thereby preventing the ingestion of potentially toxic compounds [246,247,248]. Generally, insect antifeedants possess qualities that are highly desirable for environmentally friendly crop protection agents, as they can be effective at low concentrations and their ability to target specific insect pests without harming beneficial insects or other species [249,250]. Additionally, the likelihood of insect species developing heritable resistance to these antifeedants is considered low [250,251]. Being of natural origin, these compounds are not expected to persist in the environment, offering reduced toxicity compared to synthetic pesticides and a more targeted bioactivity against specific insect pests [252,253,254]. Consequently, the investigation of insect antifeedants as crop protectants has garnered significant attention from researchers [255,256,257,258]. Unlike conventional pesticides, the active components in antifeedants do not directly cause pest mortality. Instead, they work by inducing starvation or making pests more susceptible to predation by their natural enemies. Therefore, innovative strategies are required to effectively utilize antifeedants in the field under varying environmental conditions [256].

Studies have shown that drimenol can be synthesized from drimenyl pyrophosphate through fermentation processes. This compound can subsequently be converted into antifeedants, such as warburganal and 9-hydroxydrimenal, using cost-effective methods [59,259]. Montenegro et al. (2013) aimed to identify more potent compounds derived from DW, some of which exhibited feeding dissuasive activity against Droshophila melanogaster larvae in choice assays. Drimenol demonstrated antifeedant effects, even at concentrations as low as 5 ppm, with an inhibition rate of 51.82%. An antifeedant index above 75% is typically considered significant, while moderate inhibition falls within the 50% to 75% range. Larvae treated with drimenol showed moderate feeding inhibition compared to the control, as evidenced by the reduced weight at lower concentrations of drimenol [128].

Current research on drimenol anti-insect activity reveals its potential as a bio-insecticide, but highlights several gaps. Although drimenol has demonstrated insecticidal and antifeedant properties, its mechanisms of action remain poorly understood, particularly in terms of its impact on various insect species and resistance development. Moreover, the effectiveness of drimenol in diverse environmental conditions and its safety profile for non-target organisms have not been thoroughly investigated. Future research should focus on elucidating the molecular targets of drimenol in insects, optimizing its formulation for different pest species, and conducting comprehensive ecological impact assessments to ensure its viability as a sustainable alternative to synthetic pesticides.

### 3.4. Antiparasitic Activity of Drimenol

Chagas Disease (CD) is a severe and potentially fatal condition caused by the protozoan parasite *Trypanosoma cruzi*, transmitted by blood-feeding triatomine insects from the Reduviidae family [260]. In addition to an estimated 6–8 million people currently infected and approximately 50,000 deaths annually, 65–100 million individuals reside in regions at risk of infection [261,262]. The only drugs available for CD treatment, nifurtimox and benznidazole, have been in use since the 1960s [263]. However, these medications can cause severe side effects [264]. Moreover, nifurtimox has been discontinued in several countries due to its toxicity [265]. As a result, there is an urgent need for effective anti-*Trypanosoma* compounds with lower toxicity, driving the exploration of natural products as potential new drug candidates [266,267,268]. Muñoz et al. [269,270,271] assessed the in vitro activity of several Chilean plant extracts against the trypomastigote forms of *Trypanosoma cruzi*. Drimenol was isolated from active DW extract, demonstrating activity comparable to nifurtimox and benznidazole, with an IC50 value of 25.1 µM against *Trypanosoma cruzi* trypomastigotes [269,270,271].

Parasitic helminths remain a significant concern in human and veterinary medicine, as well as in agriculture [272,273]. Approximately one-third of the global human population, particularly in developing regions, is infected with one or more nematodes, affecting over 2 billion people [274]. Intestinal nematode parasites, such as *Ascaris lumbricoides* and *Trichuris trichiura*, can cause a range of symptoms, including intestinal disturbances, systemic discomfort, and weakness, which can impair physical development and hinder the ability to work and study [275,276,277]. In addition, parasitic nematodes contribute significantly to economic losses in the livestock and crop industries worldwide. In the United States alone, nematodes account for an estimated USD 2 billion in annual losses in the livestock industry, due to reduced productivity and increased operational costs [278]. In the absence of vaccines against intestinal nematodes, chemotherapy remains the primary method of controlling infections. However, resistance to anthelmintics has been widely reported among livestock parasites and occasionally in human parasites, with the potential for becoming more common in human infections [272,279,280,281,282]. This situation underscores the urgent need for new anthelmintic compounds with novel mechanisms of action [283]. Medicinal plants offer a promising source of effective anthelmintic drugs due to their traditional use, demonstrated efficacy, and safety [284]. Anthelmintic metabolites derived from these plants may serve as potential drug candidates [285]. Some natural products from medicinal plants have shown efficacy against nematodes in both in vitro and in vivo models of *Trichuris muris* and *Schistosoma mansoni* [286,287,288]. However, many effective compounds from medicinal plants have yet to be identified. The free-living nematode *Caenorhabditis elegans* has proven to be a valuable model for discovering new anthelmintic drugs and elucidating their mechanisms of action or resistance [285,289,290,291]. Due to its ease of maintenance in the laboratory, small size, and short generation time, *Caenorhabditis elegans* is well-suited for testing the anthelmintic effects of crude plant extracts or pure compounds without the need for host infection experiments [292,293,294]. Liu et al. [295] utilized *Caenorhabditis elegans* as a model system to identify novel anthelmintic compounds from medicinal plants by assessing the motility of the nematode.

As polygodial exhibited potent activity against *Caenorhabditis elegans*, Liu et al. [295] investigated the anthelmintic activity of 18 polygodial analogs to elucidate the structural features that influence their bioactivity. Most of these analogs contained the drimane sesquiterpene skeleton. The findings revealed that drimenol had potent activity, with an IC50 value of 49.1 ± 10.1 μM [295]. Thus, drimenol may be a promising candidate for anthelmintic agent development and warrants further exploration.

Current research on drimenol antiparasitic activity shows promise but has significant gaps. While it exhibits notable in vitro effects against *Trypanosoma cruzi* and nematodes, its in vivo efficacy, safety, and pharmacokinetics are not well understood. Additionally, the mechanisms of its antiparasitic action remain unclear. Future research should focus on in vivo evaluations and uncovering its molecular targets and resistance mechanisms to improve its potential as an antiparasitic treatment.

### 3.5. Cytotoxic Activity of Drimenol

Cytotoxicity refers to the extent to which a chemical compound or substance can damage or destroy cells [296]. The toxicity of a compound to cells typically depends on extrinsic factors, including its physiochemical properties, such as structure, shape, surface, size, solubility, aggregation, and chemical nature. Toxic compounds can compromise cell membranes, leading to reduced cell viability and proliferation [296,297]. Therefore, investigating the cytotoxic properties of compounds is valuable for screening and preliminary assessing their biological properties [298].

Numerous studies have documented the cytotoxic effects of drimenol. Kahlos et al. [299] investigated the volatile constituents of *Gloeophyllum odoratum*. Using GC and GC-MS analyses, they identified the primary volatiles as linalool, citronellol, geraniol, and drimenol. These volatile oils demonstrated toxicity to brine shrimp larvae (*Artemia salina*), suggesting potential insecticidal and cytotoxic properties [299]. Moreover, Montenegro et al. [300] proposed that drimenol exhibits diverse biological activities, including antifeedant, cytotoxic, antibacterial, and antifungal effects. Similarly, Melo et al. [301] reported the multifaceted properties of drimenol, derived from *Canellaceae* species, which exhibited anti-inflammatory, cytotoxic, antifungal, and antibacterial effects [301].

Additionally, Mahnashi et al. [302] identified various compounds from *Polygonum hydropiper*, including warburganal and drimane-type sesquiterpenoids, such as confertifolin, drimenol, isopolygodial, polygodial, and isodrimeninol, which were found to possess cytotoxic properties [302]. Some researchers assert that drimenol, present in various species of the *Polygonum* (*Polygonaceae*) and *Drimys* (*Winteraceae*), is characterized by a bicyclic farnesane-type skeleton. This structural feature contributes to its diverse biological activities, which include antifungal, antibacterial, and cytotoxic effects [23,125].

The research suggests that antibacterial properties are often associated with cytotoxic effects [303]. As mentioned above in Section 3.2 (antibacterial), since drimenol possesses antibacterial properties, it is unsurprising that it also exhibits cytotoxic effects.

Current research on drimenol cytotoxic activity reveals a promising spectrum of effects, yet significant gaps remain. While drimenol has demonstrated cytotoxicity across various cell types and biological systems, the mechanisms underlying its cytotoxic action are not well understood. Additionally, studies have often focused on broad assessments of drimenol activity, without detailed investigations into its selectivity and potential off-target effects. Future research should prioritize elucidating the specific cellular pathways and molecular targets affected by drimenol to better understand its cytotoxic mechanisms.

### 3.6. Anticancer Activity of Drimenol

Cancer is a progressive disease characterized by uncontrolled and abnormal cell proliferation [304]. Annually, cancer accounts for approximately 9.8 million deaths globally, making it the second leading cause of death worldwide [304,305]. Extensive research has focused on developing natural medicines for cancer treatment, leading to the discovery of several anticancer drugs derived from medicinal plant compounds, such as podophyllotoxin, vinca alkaloids, and taxanes [304]. Drimenol is a very interesting plant secondary metabolite with tremendous biological activities [22], with anticancer activity being particularly noteworthy. Given the global burden of cancer, the cytotoxicity of drimenol against various cancer types has garnered considerable attention from natural product chemists. This section reviews recent studies on the cytotoxic effects of drimenol against cancer cells.

#### 3.6.1. In Vitro Study

Russo et al. [125] investigated the effects of an ethyl acetate extract from the bark of DW, which includes sesquiterpenoids such as polygodial, isonordrimenone, nordrimenone, and drimenol, on human melanoma cells. Drimenol treatment resulted in a significant decrease in cell viability in A2058 and A375 melanoma cells, with IC50 values of approximately 33.50 ± 0.03 μM and 31.25 ± 0.05 μM, respectively. Notably, drimenol showed no cytotoxic effects on normal human buccal fibroblasts at higher concentrations. Furthermore, drimenol (12–25 μM) induced DNA damage in A375 cells in a dose-dependent manner. The TUNEL assay confirmed that treatment with drimenol (12–25 μM) for 72 h led to a significant increase in green fluorescence indicative of DNA fragmentation. Additionally, drimenol treatment resulted in the reduced expression of the heat shock protein Hsp70 in cancer cells. Drimenol (25 μM) was also found to inhibit the anti-apoptotic protein Bcl-2, activate the pro-apoptotic protein Bax, and increase caspase-9 levels. These findings suggest that drimenol downregulates Hsp70 expression and may play a role in the apoptotic process, indicating its potential as a drug candidate for combination therapy in melanoma treatment [125].

#### 3.6.2. In Vivo Study

Essential oil from *Siparuna guianensis*, which contains 13.7 ± 0.2% drimenol, demonstrated antitumoral activity. Treatment with this essential oil resulted in a significant reduction in tumor cell counts (59.76 ± 12.33) compared to the untreated control group (96.88 ± 19.15). Additionally, the essential oil decreased MDA levels and increased SOD levels in liver tissue. These results suggest that the essential oil, due to its drimenol content, exhibits both antitumor and antioxidant properties by mitigating oxidative stress [306].

#### 3.6.3. Anticancer Mechanisms of Drimenol

**Figure 7:**
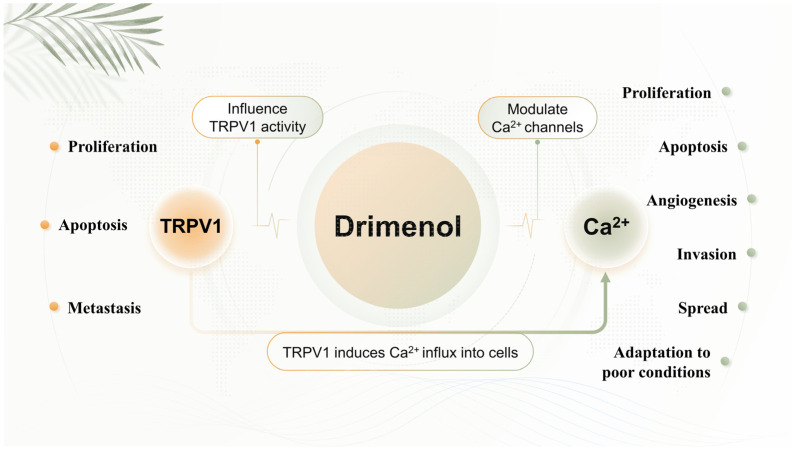
Anticancer mechanisms of drimenol.

Researchers have extensively explored the anticancer mechanism of drimenol (Figure 7). The transient receptor potential cation channel subfamily V member 1 (TRPV1) is a transmembrane protein that can be activated by various physical and chemical stimuli related to pain transduction. Recent findings have highlighted the significant roles of TRPV1 in cancer tumorigenesis and progression, as its expression levels are altered in various cancer cell types. Numerous studies have identified direct links between TRPV1 and cancer cell proliferation, apoptosis, and metastasis. Consequently, there is increased interest in examining the impacts of TRPV1 agonists and antagonists on cancer development. Both types of compounds may exhibit anticancer effects, either through TRPV1 or via alternative mechanisms [307]. Natural compounds that influence TRPV1 activity include dialdehyde terpenes, such as polygodial and drimenol [308].

Additionally, the modulation of Ca^2+^ signaling in cancer cells has emerged as a novel therapeutic target [309]. During carcinogenesis, Ca^2+^ signaling is significantly altered, disrupting normal physiological functions and conferring advantages that facilitate uncontrolled proliferation, resistance to apoptosis, angiogenesis, and adaptation to nutrient-poor conditions. These changes also enhance the ability of cells to invade and metastasize [310]. The literature suggests that plants are a valuable source of phytochemicals that may combat cancer through targeted modulation of Ca^2+^ signaling. Numerous plants with bioactive compounds have been identified as inhibitors of tumor progression and development [311]. Studies have shown that various natural ligands, including drimenol, can modulate Ca^2+^ channels [312].

These research perspectives are complementary rather than contradictory. TRPV1, like the related TRPV2–TRPV4 channels and other transient receptor potential (TRP) channels, features a pore that is non-selective for cations and exhibits significant permeability to Ca^2+^ [313]. The activation of the TRPV1 channel induces a flux of Ca^2+^ ions into cells. Intracellular Ca^2+^ overload leads to cell death [314].

Drimenol shows potential as an anticancer agent, but research gaps remain. While it exhibits anticancer effects through TRPV1 modulation and Ca^2+^ signaling interference, its impact on different cancer types of cells is not fully explored. Future studies should focus on detailed mechanisms of action, toxicity profiles, and potential synergy with other treatments to better assess drimenol therapeutic potential in clinical settings.

### 3.7. Antioxidant Activity of Drimenol

Natural antioxidants inhibit the propagation of free radical reactions, thereby protecting the human body from diseases and slowing the oxidative rancidity of lipids in food. This role helps to replace potentially harmful synthetic additives [315]. Consequently, the search for natural antioxidants is of significant importance. The antioxidant activity of drimenol has been explored in recent studies. One study analyzed the principal components of Siparuna guianensis essential oil, which include curzerenone (16.4 ± 1.5%), drimenol (13.7 ± 0.2%), and spathulenol (12.4 ± 0.8%). This oil demonstrated antioxidant activity by inhibiting 11.1 % of DPPH radicals (95.7 mg TE/g) and 15.5 % of β-carotene peroxidation [306]. Numerous other researchers shared the same perspective, asserting that drimenol exhibited antioxidant properties [316,317].

However, contrasting results were found in another study. The researchers detected 39 constituents within the essential oil extracted from *Cinnamodendron dinisii*, with drimenol comprising 0.2% of the oil. This oil showed low antioxidant activity in the β-carotene/linoleic acid test and was not detectable in the DPPH test [55]. The variability in drimenol antioxidant activity across studies may be attributed to differences in drimenol concentration and extraction methods [318]. To address these gaps, future research should focus on standardizing drimenol extraction and testing methods, as well as investigating its antioxidant mechanisms in diverse biological systems and potential interactions with other antioxidants.

### 3.8. Other Activities of Drimenol

Studies have investigated additional pharmacological properties of drimenol. Burgos et al. [319] reported that drimenol has potential as a molecular scaffold in drug development for inflammatory vascular diseases. Specifically, drimenol at a concentration of 10 μg/mL inhibited vascular cell adhesion molecule 1 and intercellular adhesion molecule 1 and reduced the adhesion of monocytes cells (THP1s) to human umbilical vein endothelial cells (HUVECs). Eser and Yoldas found that drimenol had anti-inflammatory, anti-allergic, and antiasthmatic effects [42]. In another report, drimenol, the main constituent of *Warburgia salutaris* bark, was used to treat skin and respiratory ailments [320].

## 4. Derivatives of Drimenol

Natural products and their derivatives are crucial sources for the development of new drugs [321]. In the past two decades, over 70% of new drugs have been derived from specialized plant metabolites [322]. Between 1981 and 2018, natural products and their synthetically modified analogs constituted approximately 70–80% of bioactive agents used in clinical settings [323]. Therefore, chemically modifying natural products to enhance their biological profiles and address pharmacokinetic issues, such as poor solubility, is highly advantageous [324,325,326]. Natural products are instrumental in identifying new scaffolds with diverse biological activities that can be applied directly [327,328].

Sesquiterpenes, among the most prevalent secondary metabolites derived from plants, have demonstrated significant medicinal value through extensive basic research and clinical applications. Previous studies have highlighted that sesquiterpenes and their derivatives exhibit substantial therapeutic potential against various cancers and are frequently investigated as drug candidates in clinical trials to replace traditional chemotherapeutics [28,329,330].

Drimenol, with its numerous chiral centers, including drimenol polymers, exhibits a wide range of activities and stereoselectivities. As a fundamental framework for active lead compounds, drimenol can enhance structural diversity and serve as a reference for the development of small-molecule drugs. Consequently, research efforts have focused on drimenol and its natural and synthetic derivatives to identify compounds with improved pharmacological properties. Table 2 provides an overview of the findings from different studies on drimenol derivatives, highlighting their biological activities and applications.

## 5. Conclusions and Perspectives

Naturally occurring compounds have emerged as crucial reservoirs for novel drug development. Recently, they have attracted increased attention due to their proven therapeutic efficacy and minimal toxicity, as demonstrated by compounds such as artemisinin and paclitaxel [390]. Drimenol, a significant natural compound obtainable from various herbal medicines, like PM and DW, stands out in this regard. This study provides a comprehensive overview of drimenol, covering its structure, chemical properties, plant origins, synthesis, derivatives, and biological activities. It shows that drimenol has various biological activities, including antioxidant, antifungal, antibacterial, insecticide, antiparasitic, cytotoxic, and anticancer activities. The summarized information could be valuable for guiding future research and development efforts related to drimenol.

Certainly, the journey toward the practical application of drimenol is still in progress. In the future, we should pay attention to several aspects of the research on drimenol. On the one hand, for the application of drimenol, we still need to conduct a large number of experiments, including in vitro and in vivo studies, to explore and confirm its diverse bioactivities, such as different cancer cell lines, microbial strains, and insect species, to identify its most promising therapeutic applications. Further studies are necessary to fully investigate its mechanism of action and understand its beneficial effects in humans. Elucidating the molecular mechanisms of drimenol action through advanced techniques, like omics, is essential for understanding its effects at the cellular and molecular levels. In particular, animal research and clinical trials must be encouraged. On the other hand, alongside its biological activities, ensuring the safety of drimenol remains a crucial factor constraining its potential applications. Despite reported beneficial effects, as a sesquiterpenoid, there is a need to carefully consider its potential toxicity to both humans and the environment. Presently, there have been no apparent signs of toxicity associated with drimenol. However, toxicity assessments have been limited to cellular studies. Rigorous in vivo experiments are imperative to comprehensively assess any potential side effects or toxicity, thus ensuring its safe utilization. Moreover, the absence of studies on the pharmacokinetics and bioavailability of drimenol poses a limitation. However, approaches like nanotechnology or microparticle delivery systems, which have proven effective in overcoming the poor bioavailability challenges of natural alkaloids such as berberine, could offer potential solutions to this limitation [391,392]. This includes the development of advanced drug delivery systems, such as nanoparticles and liposomes, to enhance bioavailability and ensure targeted delivery [393,394,395]. Moreover, chemical modification and drug combinations represent viable strategies for enhancing the bioavailability of bioactive compounds [396,397]. In summary, drimenol exhibits a broad spectrum of biological activities. To advance its development and applications, future research should focus on the following areas: (1) conducting additional studies to further elucidate its biological activities; (2) applying in vivo and in vitro studies to clarify the molecular mechanisms underlying its effects; (3) investigating its pharmacokinetics and bioavailability to assess biological activities; and (4) performing comprehensive toxicity assessments to determine its safety profile. By focusing on these research directions, drimenol’s promising bioactivities can be effectively translated into safe and effective clinical applications.

## Figures and Tables

**Table 1:** Main plant sources of drimenol.

Family	Plant Species	Used Part	Contents	References
Amaranthaceae	*Gomphrena celosioides*	Leaves	1.62%	[40]
Anemiaceae	*Anemia tomentosa* (Savigny) Sw. var.	Aerial parts	0.20%	[41]
Apiaceae	*Ferula elaeochytris* Korovin	Roots	0.96%	[42]
Asparagaceae	*Polygonum odoratum*	Leaves	0.39%	[43]
		Leaves	0.39%	[44]
Asteraceae	*Achillea santolina*	Aerial parts	2.33%	[45]
	*Anaphalis triplinervis*	Aerial parts	0.60%	[46]
	*Anthemis werneri* L.	\	0.10%	[47]
	*Artemisia parviflora*	Aerial parts	4.31%	[48]
	*Chrysanthemum morifolium* Ramat.	Roots	0.85%	[49]
	*Hetichrysum odoratissimum*	Leaves	0.70%	[50]
	*Ligularia fischeri* Turcz	Roots	0.09–1.33%	[51]
Canellaceae	*Canella winterana*	Leaves	\	[52]
		Leaves	4.70%	[53]
	*Capsicodendron dinisii*	Stem bark	0.70%	[54]
	*Cinnamodendron dinisii*	Leaves	0.20%	[55]
	*Warburgia salutaris*	Leaves	0.70%	[56]
		\	\	[57]
	*Warburgia ugandensis*	Bark or other parts	\	[58]
		Heartwood	\	[59]
Caprifoliaceae	*Valeriana angustifolia* Tausch	Seeds	\	[60]
	*Valeriana celtica* ssp. norica Vierh.	Seeds	\	[60]
	*Valeriana hardwickii* var. arnottiana	Roots	5.40%	[61]
	*Valeriana officinalis* var. sambucifolia	Hairy roots	\	[62]
	*Valeriana phu* L.	Seeds	\	[60]
	*Valeriana salina* Pleijel	Seeds	\	[60]
	*Valeriana sisymbriifolia* Vahl	\	4.02–4.45%	[63]
Cistaceae	*Cistus salviifolius*	Aerial parts	0.6–1.9%	[64]
Cupressaceae	*Cupressus sempervirens*	Ground material	0.37–0.4%	[65]
	*Taiwania flousiana* Gaussen	Stem bark	0.04%	[66]
Cyperaceae	*Cyperus leavigatus*	Aerial parts	0.71%	[67]
Fabaceae	*Alhagi maurorum*	Leaves	23.20%	[68]
		Stems	0.60%	[68]
		Leaves	23.20%	[69]
	*Ononis Sicula* Guss	Aerial parts	0.32%	[70]
	*Tetrapleura Tetraptera*	Leaves	0.70%	[71]
Frullaniaceae	*Frullania muscicola*	\	\	[72]
Lamiaceae	*Salvia limbata*	Leaves	0.10%	[73]
	*Scutellaria comosa*	Roots	1.44%	[74]
	*Sideritis cretica* boiss	\	0.92%	[75]
Lauraceae	*Cinnamomum camphora*	Leaves	0.23%	[76]
Lepidoziaceae	*Bazzania fauriana*	\	\	[77]
Montiniaceae	*Kaliphora* madagascariensis	Leaves	0.70%	[78]
Moraceae	*Ficus elastica*	Leaves	1%	[79]
	*Ficus polita* Vahl	\	5.80%	[80]
Myrtaceae	*Calyptranthes concinna*	Leaves	2.60%	[81]
	*Eucalyptuc obliqua*	Leaves	6.97%	[82]
	*Eucalyptus camaldulensis* var. brevirostris	Fruits	12.35%	[83]
	*Eucalyptus obliqua*	Leaves	9.33%	[84]
	*Eugenia calycina* Cambess	Leaves	0.65–0.69%	[85]
	*Eugenia protenta*	Leaves	0.1–0.7%	[86]
	*Pimenta racemosa*	Leaves	0.01–0.03%	[87]
Pinaceae	*Pinus eldarica*	Bark	13.20%	[88]
Polygonaceae	*Calligonum polygonoides*	Stems	0.70%	[89]
		Roots	29.19–29.65%	[90]
	*Calligonum Polyoides*	Roots	29.42%	[91]
	*Persicaria hydropiper*	Flowers	\	[92]
	*Polygonum acuminatum*	Leaves	\	[93]
	*Polygonum hydropiper*	Leaves	\	[94]
		\	\	[95]
		Whole plant	7.26%	[96]
		Sprouts	4.00%	[97]
	*Polygonum hydropiperoides* var. hydropiperoides	Leaves	\	[93]
	*Polygonum lapathifolium*	Leaves	\	[93]
	*Polygonum persicaria*	Leaves	\	[93]
	*Polygonum punctatum*	Leaves	\	[93]
	*Vietnamese coriander*	Leaves	0.03%	[98]
Ranunculaceae	*Clematis chinensis* Osbeck	Roots	0.20%	[99]
Sapindaceae	*Koelreuteria paniculata*	Stem bark	16.03–16.35%	[100]
Scapaniaceae	*Diplophyllum serrulatum*	\	\	[101]
Targioniaceae	*Targionia hypophylla*	Ground material	\	[102]
Verbenaceae	*Stachytarpheta indica*	Roots, shoots, and inflorescences	0.30%	[103]
Winteraceae	*Drimys angustifolia* Miers	Fresh leaves	1.40%	[104]
		Stem bark	26.20%	[104]
		Leaves	1.20%	[105]
		Leaves	1.60%	[106]
		Branch	50%	[106]
	*Drimys brasiliensis* Miers	Leaves and stem barks	4.40%	[107]
		Leaves	9.96%	[108]
		Fresh leaves	0.4–11.3%	[104]
		Dried leaves	0.10%	[104]
		Stem bark	3.7–14.6%	[104]
		Unripe fruits	0.20%	[104]
		Leaves	0.80%	[105]
		Green leaves	9.30%	[109]
		Dried leaves	11.60%	[109]
	*Drimys granadensis*	Unripe fruits	10%	[110]
		Unripe fruits	10%	[111]
		Leaves	4.30%	[112]
	*Drimys winteri*	Leaves	\	[113]
		\	\	[114]
		Fresh fruits	0.4–0.6%	[115]
		Mild dried (green) fruits	0.49–0.81%	[115]
		Strong dried (brown) fruits	0.61%	[115]
		\	3.30%	[116]
		\	12.10%	[117]
		Stem bark	2–5.8%	[118]
		\	\	[119]
	*Tasmannia lanceolata*	Leaves	\	[120]
Zingiberaceae	*Alpinia malaccensis*	Rhizomes	0.10%	[121]
	*Curcuma longa*	\	\	[122]
	*Ginger*	Fresh ginger	0.51%	[123]
	*Zingiber roseum*	Seeds	1.30%	[124]

**Table 2:** Derivatives of drimenol and their biological activities or applications.

Name of Compound	Biological Activities/Application	References
1.3 Dioxans	\	[331]
11-Aminodrim-7-ene	Antifungal activity	[332,333]
3β/-Hydroxydrimanes	Insecticidal activity	[334,335]
3β-Hydroxy-7α,8α-epoxydrimenol	\	[336]
3β-Hydroxydrimenol	\	[336]
6α-Hydroxydrimenol	\	[336]
8-Epiambreinolide	\	[337]
8-Epipuupehedione	Antitumor and cytotoxic activities	[338,339]
8β(H)-Drimane	\	[340]
9-Epiambrox	Perfumes	[341,342]
Albicanol	Cytotoxic, fish antifeedant, antifungal, anti-inflammatory, antiaging, and antioxidant activities, and antagonistic activity against heavy metal toxicity	[161,343,344,345,346,347,348,349,350]
Ambrafuran	Perfumes	[351]
Ambraoxide	Perfumes and flavoring agents in food	[352,353]
Ambrox	Perfumes	[354,355]
Isoambrox	Perfumes	[354,356]
Cyclozonarone	Cytotoxic, cytostatic, anticancer, antileishmanial, and feeding-deterrent activities	[343,357,358,359,360,361,362]
Drimenyl acetate	Antifungal activity	[149,363,364]
Forskolin	Anti-leukemic, antiproliferative, bronchodilator, anti-allergy, and hypotensive activities, and cardiac adenylate cyclase activation	[365,366,367,368,369,370,371]
Monoaldehyde drimenal	Fungistatic and fungicidal activities	[372]
Polygodial	Antibacterial, antifungal, antifeedant, antimicrobial, antinociception, anti-inflammatory, anti-allergic, anti-leishmanial, anti-trypanosomal, antifouling biocide, gastromucosal protection, cytotoxic, and insecticidal activities	[373,374,375,376,377,378,379,380,381,382,383,384,385]
Puupehenone	Antiangiogenic, antioxidant, antimicrobial, cytotoxic, antitumor, immunomodulatory, antimalarial, antiviral, antibiotic, antiatherosclerotic, and antitubercular activities	[386,387,388]
Puupehedione	Antitumor, cytotoxic, antimicrobial, and antifungal activities	[386,389]
